# Supplementary material for: Knowledge, attitude, and practice of seasonal influenza and influenza vaccine immunization among people visiting primary healthcare centers in Riyadh, Saudi Arabia
Source: PLoS One. 2022 Apr 4;17(4):e0266440. doi: 10.1371/journal.pone.0266440 (PMC8979468; doi:10.1371/journal.pone.0266440)
Supplement: Attachment — Submitted filename: Response to Reviewers .docx [file pone.0266440.s002.docx]

Dear Editor/Editorial Office,

We would like to thank you all for the valuable information to improve our article. The table attached below will provide a summary of our responses to reviewers’ comments and journal requirement.

Regards,

| **Comments** | **Response** |
| --- | --- |
| **Journal Requirements** | |
| 1. Please ensure that your manuscript meets PLOS ONE's style requirements, including those for file naming. The PLOS ONE style templates can be found at <https://journals.plos.org/plosone/s/file?id=wjVg/PLOSOne_formatting_sample_main_body.pdf> and <https://journals.plos.org/plosone/s/file?id=ba62/PLOSOne_formatting_sample_title_authors_affiliations.pdf> | Submitted files were named according to your instruction. Also, journal style template was followed. |
| 2. We note that you have indicated that data from this study are available upon request. PLOS only allows data to be available upon request if there are legal or ethical restrictions on sharing data publicly. For information on unacceptable data access restrictions, please see <http://journals.plos.org/plosone/s/data-availability#loc-unacceptable-data-access-restrictions> | Actually, there are no legal or ethical restrictions on sharing our data publicly.  Our data available at figshare repository  <https://figshare.com/s/c49083030fc8bf9f6ed> |
| In your revised cover letter, please address the following prompts:  a) If there are ethical or legal restrictions on sharing a de-identified data set, please explain them in detail (e.g., data contain potentially identifying or sensitive patient information) and who has imposed them (e.g., an ethics committee). Please also provide contact information for a data access committee, ethics committee, or other institutional body to which data requests may be sent.  b) If there are no restrictions, please upload the minimal anonymized data set necessary to replicate your study findings as either Supporting Information files or to a stable, public repository and provide us with the relevant URLs, DOIs, or accession numbers. Please see <http://www.bmj.com/content/340/bmj.c181.long> for guidelines on how to de-identify and prepare clinical data for publication. For a list of acceptable repositories, please see <http://journals.plos.org/plosone/s/data-availability#loc-recommended-repositories>. | In our revised cover letter we added this statement: “Concerning data, there are no legal or ethical restrictions on sharing our data publicly and to be uploaded in your repository. A de-identified data of 611 subjects were provided with our submission in SPSS format (data_flu.sav).”  Actually, there are no legal or ethical restrictions on sharing our data publicly. A de-identified data of 611 subjects were provided with our submission in SPSS format (data_flu.sav).  Actually, there are no legal or ethical restrictions on sharing our data publicly. A de-identified data of 611 subjects were provided/uploaded with our submission in SPSS format (data_flu.sav). |
| **Reviewer 1** | |
| - Is there a program also for the elderly and healthcare workers? Could you explain more about the Influenza vaccination program in Saudi Arabia, the target groups, numbers of individuals and access to vaccine (public or private?) | The following sentences were briefly described the influenza vaccination program in Saudi Arabia and were added to the introduction: “The Saudi Ministry of Health (MOH) offers every year a free-of-charge influenza vaccine for anyone older than six months. Furthermore, the Saudi MOH mandates an annual intake of influenza vaccination for all healthcare providers [14, 15].” |
| - Data on literature presented from lines 112 to 221 could be transferred to the discussion section, comparing to the data you generated in the article, or make it shorter. | Your comment was also mentioned by another reviewer. Both comments were considered and actually the following was done:   - 8 new articles focus on Saudi Arabia proposed by reviewer were added to the introduction in a brief way [1 systematic review (lines 190-192), 4 recruited general Saudi population and elderly (192-206), 2 enrolled healthcare workers (lines 20-252), and 1 enrolled parents (lines 253-258)]. So the majority of articles in the introduction section were discussed this topic in similar setting and population. These studies were as follows”  1. Zaraket H, Melhem N, Malik M, Khan WM, Dbaibo G, Abubakar A. Review of seasonal influenza vaccination in the Eastern Mediterranean Region: Policies, use and barriers. J Infect Public Health. 2020 Mar;13(3):377-384. doi: 10.1016/j.jiph.2020.02.029. (lines 2. Aljamili AA. Knowledge and practice toward seasonal influenza vaccine and its barriers at the community level in Riyadh, Saudi Arabia. J Family Med Prim Care. 2020 Mar 26;9(3):1331-1339. doi: 10.4103/jfmpc.jfmpc_1011_19. 3. Sales IA, Syed W, Almutairi MF, Al Ruthia Y. Public Knowledge, Attitudes, and Practices toward Seasonal Influenza Vaccine in Saudi Arabia: A Cross-Sectional Study. Int J Environ Res Public Health. 2021 Jan 8;18(2):479. doi: 10.3390/ijerph18020479. 4. Alabbad AA, Alsaad AK, Al Shaalan MA, Alola S, Albanyan EA. Prevalence of influenza vaccine hesitancy at a tertiary care hospital in Riyadh, Saudi Arabia. J Infect Public Health. 2018 Jul-Aug;11(4):491-499. doi: 10.1016/j.jiph.2017.09.002. 5. Alotaibi FY, Alhetheel AF, Alluhaymid YM, Alshibani MG, Almuhaydili AO, Alhuqayl TA, Alfayez FM, Almasabi AA. Influenza vaccine coverage, awareness, and beliefs regarding seasonal influenza vaccination among people aged 65 years and older in Central Saudi Arabia. Saudi Med J. 2019 Oct;40(10):1013-1018. doi: 10.15537/smj.2019.11.24587. 6. Alshammari TM, Yusuff KB, Aziz MM, Subaie GM. Healthcare professionals' knowledge, attitude and acceptance of influenza vaccination in Saudi Arabia: a multicenter cross-sectional study. BMC Health Serv Res. 2019 Apr 15;19(1):229. doi: 10.1186/s12913-019-4054-9. 7. Awadalla NJ, Al-Musa HM, Al-Musa KM, Asiri AM, Albariqi AA, Majrashi HM, Alasim AA, Almuslah AS, Alshehri TK, AlFlan MA, Mahfouz AA. Seasonal influenza vaccination among primary health care workers in Southwestern Saudi Arabia. Hum Vaccin Immunother. 2020;16(2):321-326. doi: 10.1080/21645515.2019.1666500. 8. Alolayan A, Almotairi B, Alshammari S, Alhearri M, Alsuhaibani M. Seasonal Influenza Vaccination among Saudi Children: Parental Barriers and Willingness to Vaccinate Their Children. Int J Environ Res Public Health. 2019 Oct 31;16(21):4226. doi: 10.3390/ijerph16214226.  - Few studies (3) were deleted from introduction and were replaced more relevant studies (mentioned in the earlier point). These studies and the line numbers were as follows: - Simaku et al. (2019, lines 99-108 - Lines 109-117 were deleted since the theme was reflected in other paragraphs - Pefil et al. (2010), lines 184-189 - Low et al. (2016), lines 2231-239 - Parts of the studies conducted in other countries were deleted from introduction (6 studies); however, their findings were integrated in the discussion section and were compared and contrasted with our study: - Masadeh et al. (2014), lines 118-126 - Mapatanon et al. (2008), lines 127-136 - Bukhsh et al. (2018), lines 137-146 - Ricco et al. (2017), lines 147-158 - Ma et al. (2018), lines 159-169 - Ermenlieva et al. (2019), lines 170-183 |
| - Were the answers to the questions about Knowledge and Practices with multiple choices or short answers? Please explain that. | Participants’ answers for knowledge, attitudes and practices were multiple-choice questions and the participants ticked for the appropriate answers. The following sentence was added to the method section “Participants’ answers in KAP domains were multiple-choice questions, and the participants ticked for the appropriate answers.” |
| - On table 3 it is reported that 356 individuals answered yes to the question “Does the vaccine have side effects?” However, on the lines below, 414 and 398 participants stated that vaccine causes Soreness and Fever, respectively. Please, check these numbers. | Yes, you are right; there is a mistake in filling the data. Numbers in table 3 were checked and corrected. 493 out of 499 (98.8%) reported the side effects of flu vaccine, and 6 out of 499 (1.2%) reported no side effects for the flu vaccine. |
| - Please make it clearer what was the knowledge added to the field of your study compared to Sagor and AlAteeq’s (2018) Alqahtani et al.’s (2017) and study. | Comment was considered, a comparison between our finding and studies of Sagor and AlAteeq’s (2018) Alqahtani et al.’s (2017) were considered in the discussion section (paragraphs 1, 3, and 6). |
| English review: Page 1 line 11: “The study investigated the relationship…” instead of “will investigate”  Line 21 “43.7% of participants had ever received” instead of “were ever received”  Page 2, line 49: the correct is “flu epidemics” not endemics  Line 55: “influenza infections across the world affects an average of five million people” sounds better than the original sentence  Line 64: find a better reference for this sentence, a scientific paper instead of Medscape  Line 70: use “its development” instead of “their development”  Page 3 line 71-72: please reformulate the sentence “The goal of the influenza vaccine is to protect against disease and achieve high vaccination rates” The vaccine itself doesn’t have the goal of achieving high vaccination rates.  Line 77: remove the word “scare”  Page 12 line 257: change “the questionnaire was clear and shows an acceptable level of internal reliability” to “the questionnaire was clear and showed an acceptable level of internal reliability”  Line 272 Please change “3. Have you ever heard of a vaccine prevent flu?” to “3. Have you ever heard that a vaccine could prevent flu?”  Line 388: change “There were 43.7% of participants (267 out of 611) were ever received flu vaccine ” to “There were 43.7% of participants (267 out of 611) that ever received flu vaccine” | Noted, and corrected  Noted, and corrected  Noted, and corrected  Noted, proposed sentence replaced the original one  A Medscape source was replace by a primary source article “J. W. Drake, “Rates of spontaneous mutation among RNA viruses,” Proceedings of the National Academy of Sciences, vol. 90, no. 9, pp. 4171-4175, 1993.”  Noted, and corrected  Noted, statement was reformulated and the following statement was deleted “and achieve high vaccination rates”  Noted, word was removed  Noted, proposed sentence replaced the original one  Noted, statement was replaced with the new one  Noted, proposed sentence replaced the original one |
| **Reviewer 2** | |
| The manuscript titled "Knowledge, attitude, and practice of seasonal influenza and influenza vaccine immunization among people visiting primary healthcare centers in Riyadh, Saudi Arabia" by Norah Alhatim, Ahmad M. Al-Bashaireh and Ola M. Alqudah presents a cross-sectional, descriptive and correlational study to assess the knowledge, attitudes, and practices (KAP) of seasonal influenza and influenza vaccine immunization among four Riyadh’s primary healthcare centers. Through the data found, it is clear the need to invest in public awareness policies to increase population adherence in vaccination campaigns in order to mitigate potential new epidemics. The lack of basic and scientific knowledge, combined with religious factors, can contribute to vaccine-refractory attitudes. This theme, when extrapolated to the Covid pandemic, shows the importance of investing in vaccine adherence campaigns with the support of local competent authorities. | Thank you for the feedback. We totally agreed that further activities were needed to increase public awareness about seasonal influenza and influenza vaccine and it was a part of our recommendation. |
| **Reviewer 3** | |
| Major points: | |
| In my opinion the manuscript needs to be actualized, benefiting greatly with the inclusion of several studies listed in this revision (discussion section below). | Please see comment under the major points: discussion. Discussion section was revised and the majority of proposed articles were included (8 out of 11). Please check below notes under introduction and discussion. |
| - Introduction  In my opinion some paragraphs can be removed and other can be presented in a simple and lean way. Paragraphs from line 141 to 183 should be removed and the ones from lines 184 to 228 can be moved to the discussion section in a more concise form. | Comments were considered. Your comment was also mentioned by another reviewer. Both comments were considered and actually the following was done:   - 8 new articles focus on Saudi Arabia proposed by reviewer were added to the introduction in a brief way [1 systematic review (**lines 190-192**), 4 recruited general Saudi population and elderly (**lines** **192-206**), 2 enrolled healthcare workers (**lines 20-252**), and 1 enrolled parents (**lines 253-258**)]. So the majority of articles in the introduction section were discussed this topic in similar setting and population. These studies were as follows”  1. Zaraket H, Melhem N, Malik M, Khan WM, Dbaibo G, Abubakar A. Review of seasonal influenza vaccination in the Eastern Mediterranean Region: Policies, use and barriers. J Infect Public Health. 2020 Mar;13(3):377-384. doi: 10.1016/j.jiph.2020.02.029. (lines 2. Aljamili AA. Knowledge and practice toward seasonal influenza vaccine and its barriers at the community level in Riyadh, Saudi Arabia. J Family Med Prim Care. 2020 Mar 26;9(3):1331-1339. doi: 10.4103/jfmpc.jfmpc_1011_19. 3. Sales IA, Syed W, Almutairi MF, Al Ruthia Y. Public Knowledge, Attitudes, and Practices toward Seasonal Influenza Vaccine in Saudi Arabia: A Cross-Sectional Study. Int J Environ Res Public Health. 2021 Jan 8;18(2):479. doi: 10.3390/ijerph18020479. 4. Alabbad AA, Alsaad AK, Al Shaalan MA, Alola S, Albanyan EA. Prevalence of influenza vaccine hesitancy at a tertiary care hospital in Riyadh, Saudi Arabia. J Infect Public Health. 2018 Jul-Aug;11(4):491-499. doi: 10.1016/j.jiph.2017.09.002. 5. Alotaibi FY, Alhetheel AF, Alluhaymid YM, Alshibani MG, Almuhaydili AO, Alhuqayl TA, Alfayez FM, Almasabi AA. Influenza vaccine coverage, awareness, and beliefs regarding seasonal influenza vaccination among people aged 65 years and older in Central Saudi Arabia. Saudi Med J. 2019 Oct;40(10):1013-1018. doi: 10.15537/smj.2019.11.24587. 6. Alshammari TM, Yusuff KB, Aziz MM, Subaie GM. Healthcare professionals' knowledge, attitude and acceptance of influenza vaccination in Saudi Arabia: a multicenter cross-sectional study. BMC Health Serv Res. 2019 Apr 15;19(1):229. doi: 10.1186/s12913-019-4054-9. 7. Awadalla NJ, Al-Musa HM, Al-Musa KM, Asiri AM, Albariqi AA, Majrashi HM, Alasim AA, Almuslah AS, Alshehri TK, AlFlan MA, Mahfouz AA. Seasonal influenza vaccination among primary health care workers in Southwestern Saudi Arabia. Hum Vaccin Immunother. 2020;16(2):321-326. doi: 10.1080/21645515.2019.1666500. 8. Alolayan A, Almotairi B, Alshammari S, Alhearri M, Alsuhaibani M. Seasonal Influenza Vaccination among Saudi Children: Parental Barriers and Willingness to Vaccinate Their Children. Int J Environ Res Public Health. 2019 Oct 31;16(21):4226. doi: 10.3390/ijerph16214226.  - Few studies (3) were deleted from introduction and were replaced more relevant studies (mentioned in the earlier point). These studies and the line numbers were as follows: - Simaku et al. (2019, lines 99-108 - Lines 109-117 were deleted since the theme was reflected in other paragraphs - Pefil et al. (2010), lines 184-189 - Low et al. (2016), lines 2231-239 - Parts of the studies conducted in other countries were deleted from introduction (6 studies); however, their findings were integrated in the discussion section and were compared and contrasted with our study: - Masadeh et al. (2014), lines 118-126 - Mapatanon et al. (2008), lines 127-136 - Bukhsh et al. (2018), lines 137-146 - Ricco et al. (2017), lines 147-158 - Ma et al. (2018), lines 159-169 - Ermenlieva et al. (2019), lines 170-183 |
| - Discussion  As mentioned before it needs to incorporate related studies that will give the reader a better understand of your results and the published literature in similar subjects.  Discussion and introduction sections will benefit greatly from the following articles:  Aljamili AA. Knowledge and practice toward seasonal influenza vaccine and its barriers at the community level in Riyadh, Saudi Arabia. J Family Med Prim Care. 2020 Mar 26;9(3):1331-1339. doi: 10.4103/jfmpc.jfmpc_1011_19.  Alabbad AA, Alsaad AK, Al Shaalan MA, Alola S, Albanyan EA. Prevalence of influenza vaccine hesitancy at a tertiary care hospital in Riyadh, Saudi Arabia. J Infect Public Health. 2018 Jul-Aug;11(4):491-499. doi: 10.1016/j.jiph.2017.09.002.  Alotaibi FY, Alhetheel AF, Alluhaymid YM, Alshibani MG, Almuhaydili AO, Alhuqayl TA, Alfayez FM, Almasabi AA. Influenza vaccine coverage, awareness, and beliefs regarding seasonal influenza vaccination among people aged 65 years and older in Central Saudi Arabia. Saudi Med J. 2019 Oct;40(10):1013-1018. doi: 10.15537/smj.2019.11.24587.  Sales IA, Syed W, Almutairi MF, Al Ruthia Y. Public Knowledge, Attitudes, and Practices toward Seasonal Influenza Vaccine in Saudi Arabia: A Cross-Sectional Study. Int J Environ Res Public Health. 2021 Jan 8;18(2):479. doi: 10.3390/ijerph18020479.  Zaraket H, Melhem N, Malik M, Khan WM, Dbaibo G, Abubakar A. Review of seasonal influenza vaccination in the Eastern Mediterranean Region: Policies, use and barriers. J Infect Public Health. 2020 Mar;13(3):377-384. doi: 10.1016/j.jiph.2020.02.029.  Balkhy HH, Abolfotouh MA, Al-Hathlool RH, Al-Jumah MA. Awareness, attitudes, and practices related to the swine influenza pandemic among the Saudi public. BMC Infect Dis. 2010 Feb 28;10:42. doi: 10.1186/1471-2334-10-42.  Alshammari TM, Yusuff KB, Aziz MM, Subaie GM. Healthcare professionals' knowledge, attitude and acceptance of influenza vaccination in Saudi Arabia: a multicenter cross-sectional study. BMC Health Serv Res. 2019 Apr 15;19(1):229. doi: 10.1186/s12913-019-4054-9.  Awadalla NJ, Al-Musa HM, Al-Musa KM, Asiri AM, Albariqi AA, Majrashi HM, Alasim AA, Almuslah AS, Alshehri TK, AlFlan MA, Mahfouz AA. Seasonal influenza vaccination among primary health care workers in Southwestern Saudi Arabia. Hum Vaccin Immunother. 2020;16(2):321-326. doi: 10.1080/21645515.2019.1666500.  Alolayan A, Almotairi B, Alshammari S, Alhearri M, Alsuhaibani M. Seasonal Influenza Vaccination among Saudi Children: Parental Barriers and Willingness to Vaccinate Their Children. Int J Environ Res Public Health. 2019 Oct 31;16(21):4226. doi: 10.3390/ijerph16214226.  Balaban V, Stauffer WM, Hammad A, Afgarshe M, Abd-Alla M, Ahmed Q, Memish ZA, Saba J, Harton E, Palumbo G, Marano N. Protective practices and respiratory illness among US travelers to the 2009 Hajj. J Travel Med. 2012 May-Jun;19(3):163-8. doi: 10.1111/j.1708-8305.2012.00602.x.  Alqahtani AS, Rashid H, Heywood AE. Vaccinations against respiratory tract infections at Hajj. Clin Microbiol Infect. 2015 Feb;21(2):115-27. doi: 10.1016/j.cmi.2014.11.026. | Thank you so much for the references and the valuable comments. Your comment was considered. Actually the majorities of these references were included in the introduction and discussion section and were compared and contrasted with our findings.   - 8 new articles focus on Saudi Arabia proposed by reviewer were added to the introduction and discussion section in a brief way [4 recruited general Saudi population and elderly, 2 enrolled healthcare workers, and 1 enrolled parents]. So the majority of articles in the introduction section were discussed this topic in similar setting and population. These studies were as follows”  1. Zaraket H, Melhem N, Malik M, Khan WM, Dbaibo G, Abubakar A. Review of seasonal influenza vaccination in the Eastern Mediterranean Region: Policies, use and barriers. J Infect Public Health. 2020 Mar;13(3):377-384. doi: 10.1016/j.jiph.2020.02.029.   **(added to discussion lines 546-548)**   2. Aljamili AA. Knowledge and practice toward seasonal influenza vaccine and its barriers at the community level in Riyadh, Saudi Arabia. J Family Med Prim Care. 2020 Mar 26;9(3):1331-1339. doi: 10.4103/jfmpc.jfmpc_1011_19.   **(added to discussion lines 482-486)**   3. Sales IA, Syed W, Almutairi MF, Al Ruthia Y. Public Knowledge, Attitudes, and Practices toward Seasonal Influenza Vaccine in Saudi Arabia: A Cross-Sectional Study. Int J Environ Res Public Health. 2021 Jan 8;18(2):479. doi: 10.3390/ijerph18020479.   **(added to discussion lines 482-486, 503-506)**   4. Alabbad AA, Alsaad AK, Al Shaalan MA, Alola S, Albanyan EA. Prevalence of influenza vaccine hesitancy at a tertiary care hospital in Riyadh, Saudi Arabia. J Infect Public Health. 2018 Jul-Aug;11(4):491-499. doi: 10.1016/j.jiph.2017.09.002.   **(added to discussion lines 553-556)**   5. Alotaibi FY, Alhetheel AF, Alluhaymid YM, Alshibani MG, Almuhaydili AO, Alhuqayl TA, Alfayez FM, Almasabi AA. Influenza vaccine coverage, awareness, and beliefs regarding seasonal influenza vaccination among people aged 65 years and older in Central Saudi Arabia. Saudi Med J. 2019 Oct;40(10):1013-1018. doi: 10.15537/smj.2019.11.24587. 6. Alshammari TM, Yusuff KB, Aziz MM, Subaie GM. Healthcare professionals' knowledge, attitude and acceptance of influenza vaccination in Saudi Arabia: a multicenter cross-sectional study. BMC Health Serv Res. 2019 Apr 15;19(1):229. doi: 10.1186/s12913-019-4054-9.   **(added to discussion lines 560-566)**   7. Awadalla NJ, Al-Musa HM, Al-Musa KM, Asiri AM, Albariqi AA, Majrashi HM, Alasim AA, Almuslah AS, Alshehri TK, AlFlan MA, Mahfouz AA. Seasonal influenza vaccination among primary health care workers in Southwestern Saudi Arabia. Hum Vaccin Immunother. 2020;16(2):321-326. doi: 10.1080/21645515.2019.1666500.   **(added to discussion lines 560-566)**   8. Alolayan A, Almotairi B, Alshammari S, Alhearri M, Alsuhaibani M. Seasonal Influenza Vaccination among Saudi Children: Parental Barriers and Willingness to Vaccinate Their Children. Int J Environ Res Public Health. 2019 Oct 31;16(21):4226. doi: 10.3390/ijerph16214226.   **( lines 253-258)** |
| Minor points: | |
| Abstract Page 1 line 2 and 3: rephrase the following sentence: “Influenza infection is a continuing threat to the Saudi population, resulting in high mortality rates and disease prevalence; it also puts significant financial strain on the country.” | Comment was considered, sentence was rephrased as follows: “Influenza infection continues to be a hazard to the Saudi population, resulting in high death rates and illness prevalence; it also places a substantial financial burden on the government.” |
| Introduction This section needs to be shortened and actualized (please see articles suggested at the discussion section).  Page 3 line 57: remove: “However, it affects all age groups and is usually self-limited [4]” | Your comment was also mentioned by another reviewer. Both comments were considered and actually the following was done:   - 8 new articles focus on Saudi Arabia proposed by reviewer were added to the introduction in a brief way [1 systematic review (**lines 190-192**), 4 recruited general Saudi population and elderly (**lines 192-206**), 2 enrolled healthcare workers (**lines 20-252**), and 1 enrolled parents (**lines 253-258**)]. So the majority of articles in the introduction section were discussed this topic in similar setting and population. These studies were as follows”  1. Zaraket H, Melhem N, Malik M, Khan WM, Dbaibo G, Abubakar A. Review of seasonal influenza vaccination in the Eastern Mediterranean Region: Policies, use and barriers. J Infect Public Health. 2020 Mar;13(3):377-384. doi: 10.1016/j.jiph.2020.02.029. (lines 2. Aljamili AA. Knowledge and practice toward seasonal influenza vaccine and its barriers at the community level in Riyadh, Saudi Arabia. J Family Med Prim Care. 2020 Mar 26;9(3):1331-1339. doi: 10.4103/jfmpc.jfmpc_1011_19. 3. Sales IA, Syed W, Almutairi MF, Al Ruthia Y. Public Knowledge, Attitudes, and Practices toward Seasonal Influenza Vaccine in Saudi Arabia: A Cross-Sectional Study. Int J Environ Res Public Health. 2021 Jan 8;18(2):479. doi: 10.3390/ijerph18020479. 4. Alabbad AA, Alsaad AK, Al Shaalan MA, Alola S, Albanyan EA. Prevalence of influenza vaccine hesitancy at a tertiary care hospital in Riyadh, Saudi Arabia. J Infect Public Health. 2018 Jul-Aug;11(4):491-499. doi: 10.1016/j.jiph.2017.09.002. 5. Alotaibi FY, Alhetheel AF, Alluhaymid YM, Alshibani MG, Almuhaydili AO, Alhuqayl TA, Alfayez FM, Almasabi AA. Influenza vaccine coverage, awareness, and beliefs regarding seasonal influenza vaccination among people aged 65 years and older in Central Saudi Arabia. Saudi Med J. 2019 Oct;40(10):1013-1018. doi: 10.15537/smj.2019.11.24587. 6. Alshammari TM, Yusuff KB, Aziz MM, Subaie GM. Healthcare professionals' knowledge, attitude and acceptance of influenza vaccination in Saudi Arabia: a multicenter cross-sectional study. BMC Health Serv Res. 2019 Apr 15;19(1):229. doi: 10.1186/s12913-019-4054-9. 7. Awadalla NJ, Al-Musa HM, Al-Musa KM, Asiri AM, Albariqi AA, Majrashi HM, Alasim AA, Almuslah AS, Alshehri TK, AlFlan MA, Mahfouz AA. Seasonal influenza vaccination among primary health care workers in Southwestern Saudi Arabia. Hum Vaccin Immunother. 2020;16(2):321-326. doi: 10.1080/21645515.2019.1666500. 8. Alolayan A, Almotairi B, Alshammari S, Alhearri M, Alsuhaibani M. Seasonal Influenza Vaccination among Saudi Children: Parental Barriers and Willingness to Vaccinate Their Children. Int J Environ Res Public Health. 2019 Oct 31;16(21):4226. doi: 10.3390/ijerph16214226.  - Few studies (3) were deleted from introduction and were replaced more relevant studies (mentioned in the earlier point). These studies and the line numbers were as follows: - Simaku et al. (2019, lines 99-108 - Lines 109-117 were deleted since the theme was reflected in other paragraphs - Pefil et al. (2010), lines 184-189 - Low et al. (2016), lines 2231-239 - Parts of the studies conducted in other countries were deleted from introduction (6 studies); however, their findings were integrated in the discussion section and were compared and contrasted with our study: - Masadeh et al. (2014), lines 118-126 - Mapatanon et al. (2008), lines 127-136 - Bukhsh et al. (2018), lines 137-146 - Ricco et al. (2017), lines 147-158 - Ma et al. (2018), lines 159-169 - Ermenlieva et al. (2019), lines 170-183   Comment was considered, sentence was removed. |
| Materials and Methods Page 11 - Line 236-239: Informe the period (years) of data collection.  Page 12/13 - Line 262 -293: Variables and Domains should be presented in a box instead of being listed in the text. This way will be easier for the reader to have access to the information. | Years, months of data collection were added to the following sentence under the method section.  The original sentence was “Sample of 611 individuals were subsequently recruited.” and the current one is “Sample of 611 individuals were subsequently recruited between January 15 and the fifth of July 2020.”  Comment was considered, the domains and their related items were presented in Box 1. |
| Results Page 15 line 331: “When asked about the flu”  Page 15 line 337: Table 1. Socio-demographic characteristics (N = 611).  Page 18 line 362-370: Tables 4 to 7 should be merged in a unique table with all information.  Page 19: Table 9 should be excluded the information is already on the text. This Table brings no additional information to the reader, this suggestion is also applicable for Tables 11, 12 and 14 on page 20, 21 and 22, respectively. | This statement was replaced with a more clear statement “Participants’ knowledge concerning seasonal influenza revealed that”  Comment was considered, title of table 1 was changed from “Sample characteristics (N = 611)” to “Socio-demographic characteristics (N = 611)”  Comment was considered, the information in 4 tables (4-7) were reduced to 1 table (Tables 4) and still including all information.  Tables 9, 11, and 14 were removed as recommended. However; we kept Table 12 since it extra information that was not reported in the text. |
| Discussion See Major points above. | Please see comment under the major points: discussion. |
| Recommendations  It’s very important to include here proposals of how increased education regarding influenza and vaccinations can be achieved, which activities need to be better addressed and which should be strengthen. | Statements concerning activities of how to increase education regarding influenza and vaccinations were added as follows: “Along with the flu vaccine campaign, a systematic public awareness program on seasonal influenza and influenza vaccinations is advised to be undertaken yearly. Furthermore, healthcare providers were advised to discuss the importance of getting a flu vaccine on a regular basis with their patients, particularly those who were at risk.” |
| Conclusion  Page 26 - Lines 520 to 523: remove | Comment was considered, lines from 520 to 523 were removed. |
|  |  |
