# Supplementary material for: Knowledge, attitude, and practice of seasonal influenza and influenza vaccine immunization among people visiting primary healthcare centers in Riyadh, Saudi Arabia
Source: PLoS One. 2022 Apr 4;17(4):e0266440. doi: 10.1371/journal.pone.0266440 (PMC8979468; doi:10.1371/journal.pone.0266440)
Supplement: Attachment — Submitted filename: Response to Reviewers .docx [file pone.0266440.s003.docx]

Dear Editor/Editorial Office,

We would like to thank you all for the valuable information to improve our article. The table attached below will provide a summary of our responses to reviewers’ comments and editor as well as the journal requirement.

Regards,

| **Comments** | **Response** |
| --- | --- |
| **Editor Note & Journal Requirements** | |
| In your methods section, please provide additional information about the participant recruitment method and the demographic details of your participants. In particular, please include a statement as to whether your sample can be considered representative of a larger population. In addition, please further discuss as a limitation in the Results or Conclusion section the non-representativeness of your sample (in particular the relatively high rate of respondents who attended college). | Thank you for the comments. All recommended changes were done.  Concerning recruitment methods and demographics, the following paragraph was added after the first paragraph under the Materials and Methods (after line 175):  “Active recruitment was implemented in this study. Researchers contacted potential participants from the four primary healthcare centers in Riyadh city (Alsylimania, Alwady, Alyasmin, and Alsahafah). They explained the purpose, confidentiality, anonymity, and voluntariness of participating in this study. Potential participants were anyone who visited these four primary healthcare centers 18 years old and above, can read and write the Arabic language, including patients or their companions (e.g., parents of young children and family caregivers of elderly patients). If participants gave their verbal consent, they were asked to fill a self-reported questionnaire that includes demographics, knowledge, and attitude concerning seasonal influenza and influenza vaccination. Participants took a time of 10 to 15 minutes to complete the self-reported questionnaire.”  Concerning generalization due to the sample representativeness, the following sentence was added to the limitation under the discussion section (line 433):  “Also, because most participants were male, employed, held college or university degrees, extra caution should be exercised in extrapolating our findings to the entire Saudi population.” |
| PLOS ONE does not copy edit accepted manuscripts. Please proofread for typos and grammar, including in the abstract. | The comment was considered. After implementing the recommended changes, the manuscript was gone over proofread services via an authenticated company. The prove of editing services was attached with this message. |
| **Reviewer 1** | |
| I think the review answered all the questions and points I had raised and the new version has been improved substantially. Just another question that arose when I read the reviewed manuscript. The data collection for this study was from January to July 2020. It would be important to include one or two sentences discussing the impact of Covid-19 pandemics on the results obtained in this study, compared to previous years, without the threat of a respiratory pandemic. | The comment was considered, the following sentences were added to the limitation under the discussion section (line 435):  “Unlike earlier studies, this study was conducted during a respiratory pandemic caused by Corona Virus Disease-2019 (COVID-19). This may bias the results in favor of certain directions. Moreover, the cross-sectional design of the study precludes the causality.” |
|  | |
